# A Rare Case Report: Five Variants of Lichen Planus in a Young Male Patient

**DOI:** 10.7759/cureus.27080

**Published:** 2022-07-20

**Authors:** Fatema A Al Khabbaz, Mahmood M Ali, Ameen Al Awadhi

**Affiliations:** 1 Dermatology, Salmaniya Medical Complex, Manama, BHR

**Keywords:** variants, pruritis, bullous lichen planus, : lichen planus pigmentosus, classic lichen planus, licheniod, lichen planus

## Abstract

Lichen planus is a common dermatological condition. It is described as a chronic inflammatory mucocutaneous disease that has characteristic clinical and histopathological findings. Classical lichen planus lesions occur as purple, pruritic polygonal papules or plaques with a lace-like pattern of whitish markings on the surface. Despite the large number of variants that exist with different clinical manifestations from the classic form, histopathology features are fairly similar among the subtypes and can aid in the diagnosis. Although most cases of lichen planus are often self-limiting with spontaneous resolution expected within one to two years, early diagnosis and treatment are encouraged to control severe pruritus and painful mucosal erosion, but most importantly to minimize the potential for malignant transformation in long-standing lesions. The main objective of this paper is to report the first case of five cutaneous variants of lichen planus occurring simultaneously in a young male patient.

## Introduction

Lichen planus is a common inflammatory mucocutaneous disease affecting around 1% of the world's population. The diseases can be cutaneous, mucosal, or mucocutaneous [1].

Lesions typically present as purple, pruritic polygonal papules or plaques with a lace-like pattern of whitish markings on the surface (known as Wickham striae). The lesions are commonly symmetrical and can affect any part of the body surface, but they do favor the flexural surfaces of the forearms, wrists, ankles, dorsum of the hands, shins, trunk, and sacral region [2,3]. Lichen planus can also involve the scalp, hair, and nails [2]. Oral involvement is also common; around 50% of patients with cutaneous lesions have oral lesions, whereas around 25% of patients will present with oral lesions only. In contrast to cutaneous disease, oral lesions tend to be more chronic [1].

Although classic lichen planus is a common disease, there are very few reported cases of three or more forms of lichen planus occurring simultaneously in an individual [4]. Here we present a case of cutaneous lichen planus occurring in five different forms: classic, hypertrophic, annular, bullous, and pigmentosus, all in one patient at the time of presentation.

## Case presentation

A 25-year-old Middle Eastern male was referred to our Dermatology clinic with complaints of pigmented lesions scattered over his upper limbs, underarms, and lower limbs, associated with mild pruritus.

The lesions appeared one year prior and progressed in size and count. The patient also complained of lesions over his upper limbs, elbows, and feet, which on examination were found to be hypertrophic papules with associated pruritus. At the time of presentation, the patient’s lesions were inadequately controlled by mometasone furoate 0.1% ointment, which he had been using once a day intermittently for the past year. The patient had been following up with different dermatologists prior to presenting to our clinic, but he couldn’t find a solution and kept on getting new types of lesions. That’s why the patient was referred to a tertiary health care facility to address this polymorphous rash. He had not received any systemic treatments or phototherapy up until that point. The patient denied having a family history of similar lesions or any other dermatological disease. He denied taking any medication prior to the eruption, making lichenoid drug eruption less likely.

**Figure 1:**
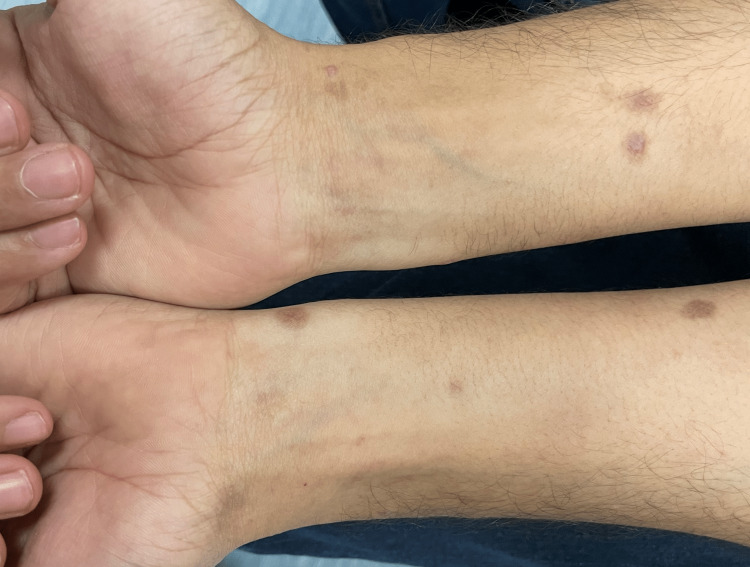
Multiple violaceous papules and plaques, admixed with grey brown macules on the wrist and forearms, indicate the classic type of lichen planus.

**Figure 2:**
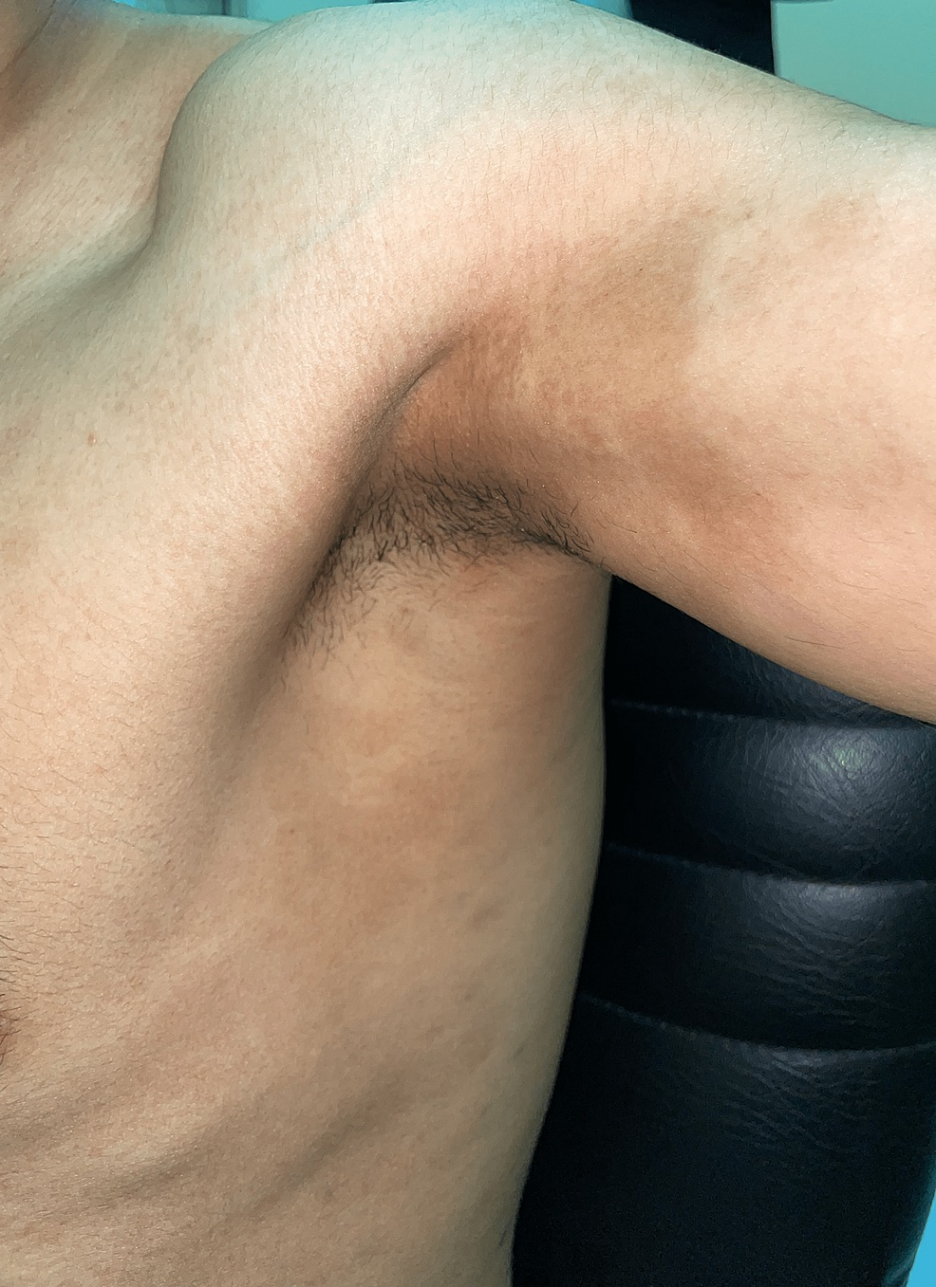
Greyish brown macules and patches over the flexural sites representing the pigmentosus variant.

**Figure 3:**
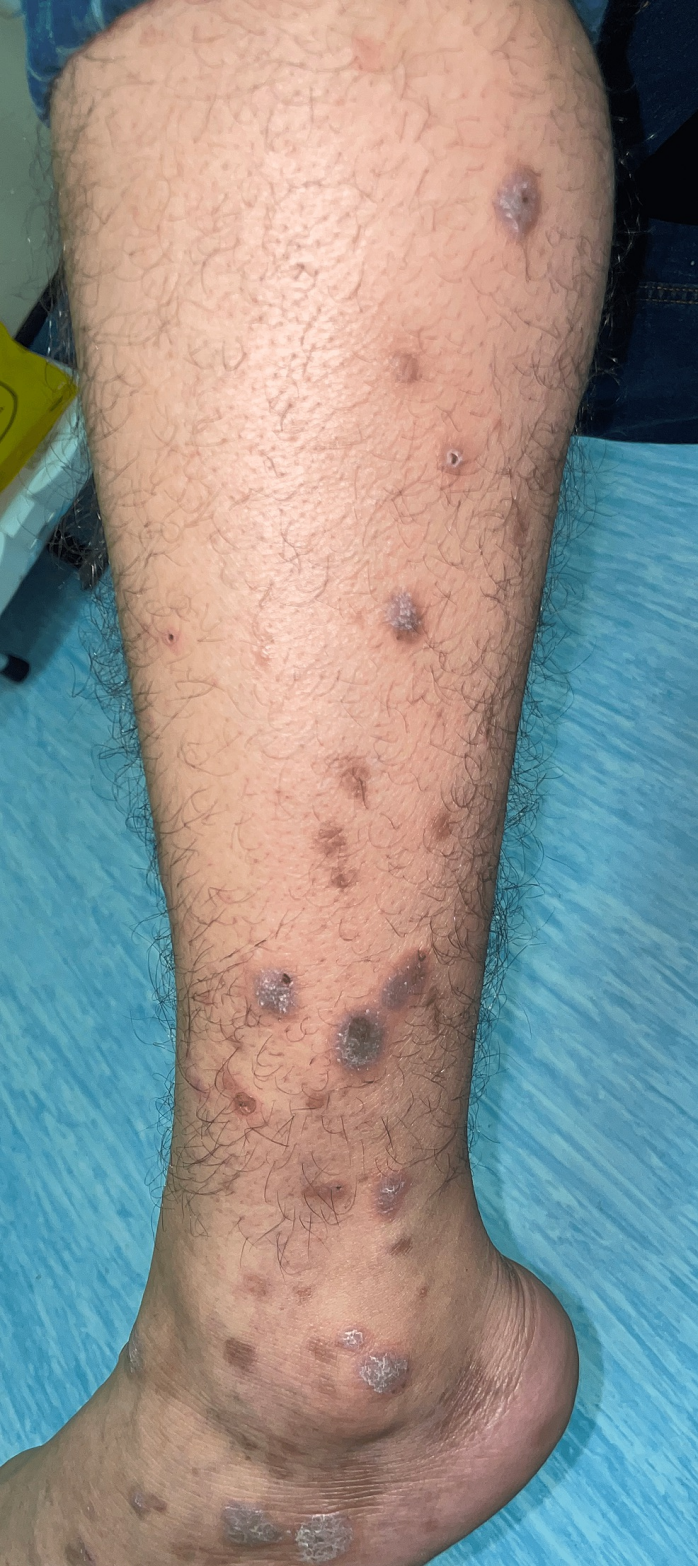
Grayish purple papules and plaques on the pretibial area of the lower extremity and the ankle, representing the hypertrophic type.

**Figure 4:**
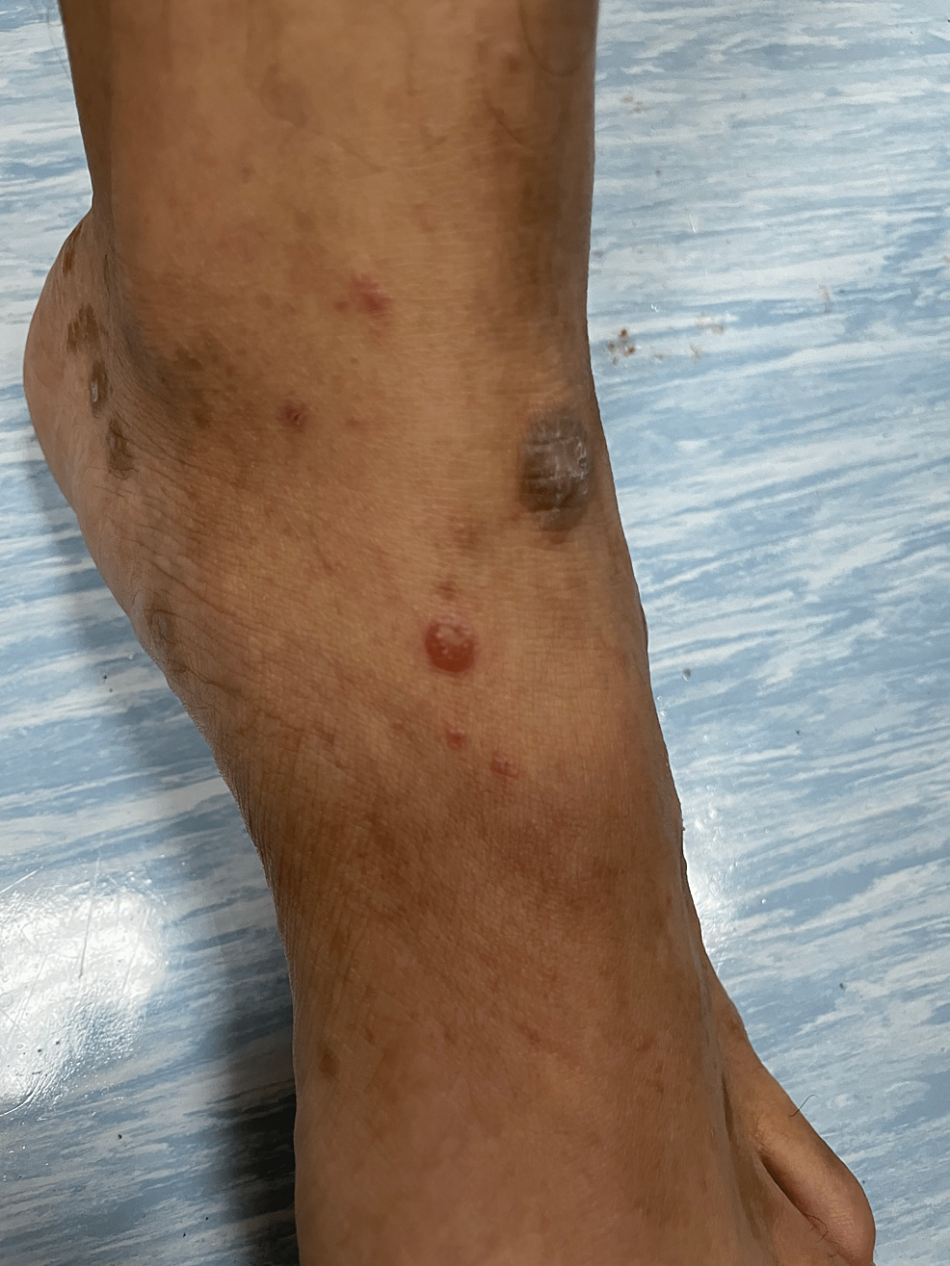
Tense bullae were noted on the feet representing the bullous type of lichen planus.

**Figure 5:**
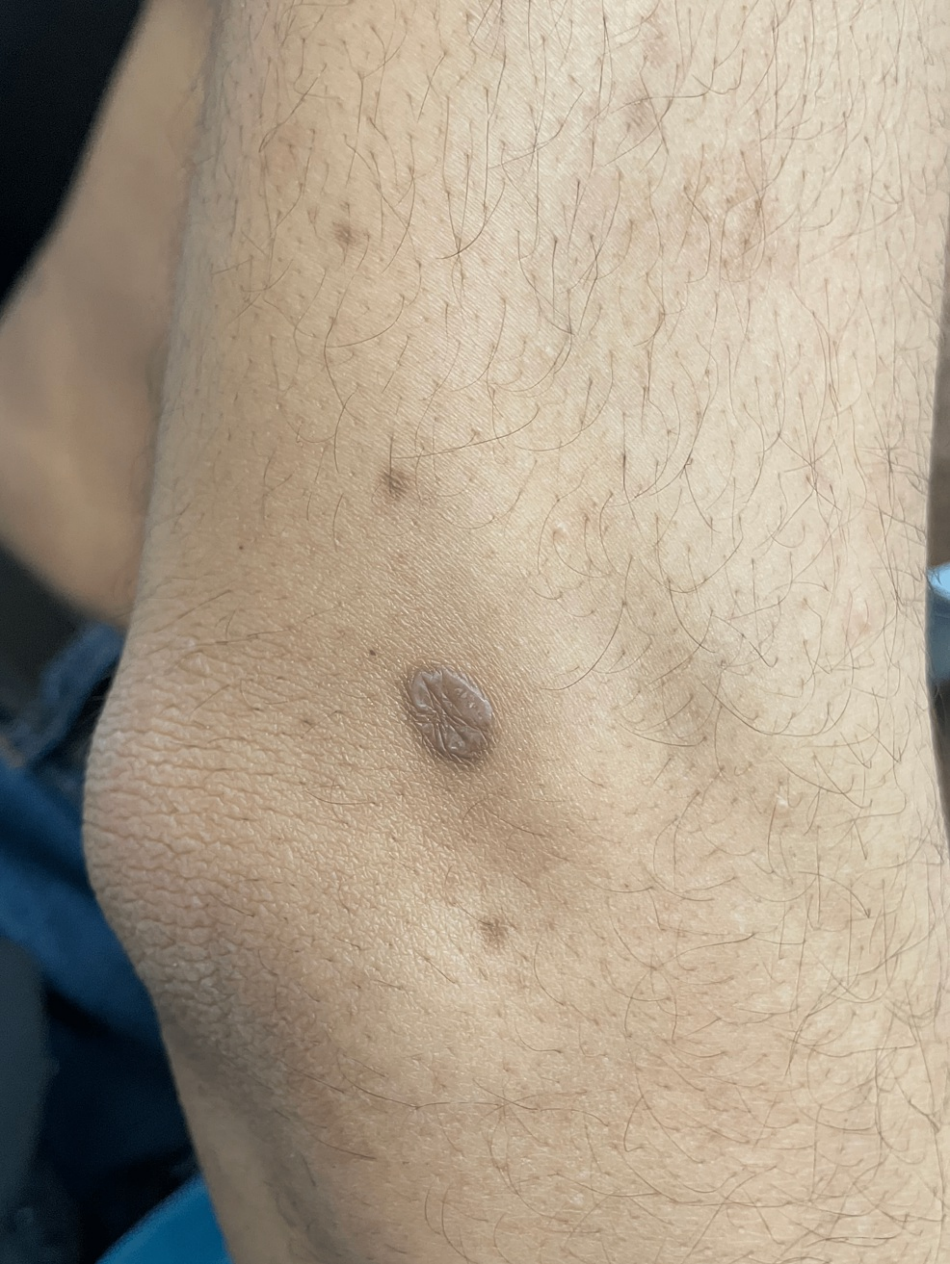
Annular lesions were noted on the patient's elbows.

**Figure 6:**
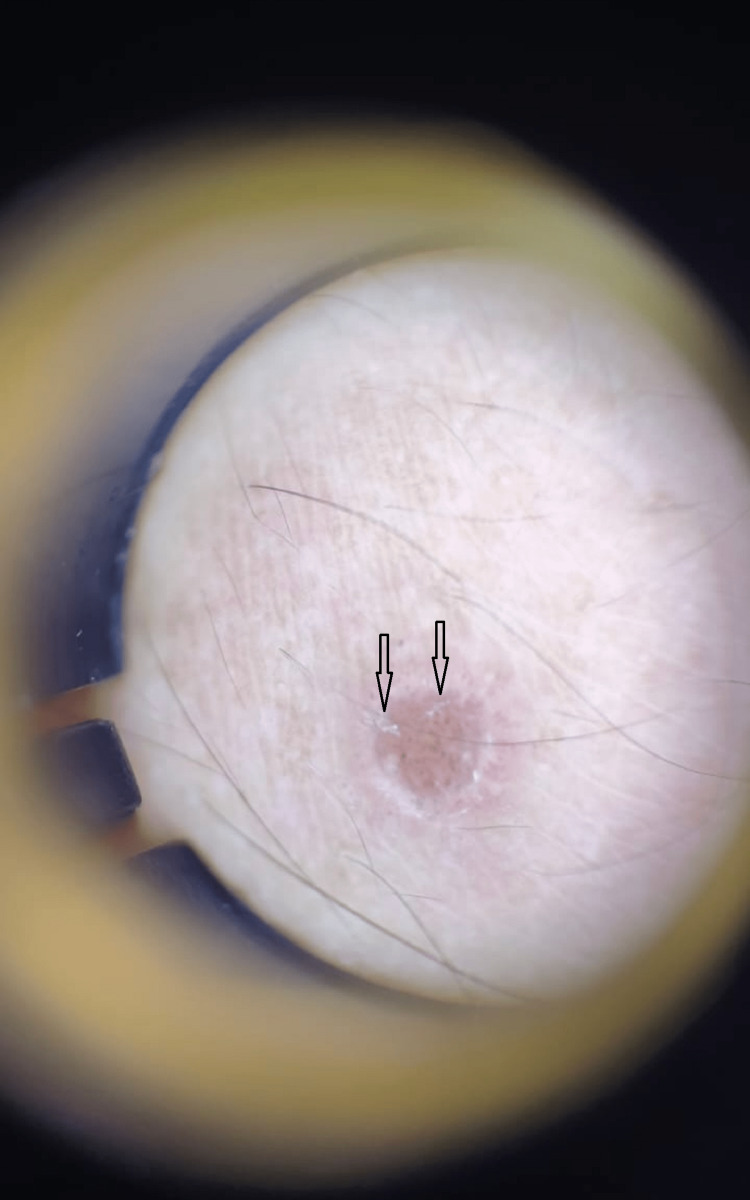
Dermatoscopy examination of the lesions showing Wickham striae clearly. Black arrows: Wickham striae

On clinical examination, there were multiple violaceous papules and plaques, admixed with greyish brown macules, on the wrist and forearms, indicating the classic type of lichen planus (former) and its post-inflammatory hyperpigmentation (latter) (Figure 1). The flexural sites revealed greyish brown macules and patches representing the pigmentosus variant (Figure 2). The patient also had grey purple hypertrophic papules and plaques on the pretibial area of the lower extremity and the ankles, representing the hypertrophic type (Figure 3). Also, tense bullae admixed with classic lichen planus lesions were noted on the feet (Figure 4), and annular lesions were noted on the patient's elbows (Figure 5). The lesions were examined by dermatoscopy, in which Wickham striae were clearly seen (Figure 6). However, examination of the oral mucosa, hair, nails, and genital mucosa did not reveal any features or other forms of lichen planus.

The differential diagnosis after taking a detailed history and a focused physical examination, including the use of dermatoscopy, was: lichen planus with multiple variants, ashy dermatosis, mycosis fungoides, and psoriasis.

**Figure 7:**
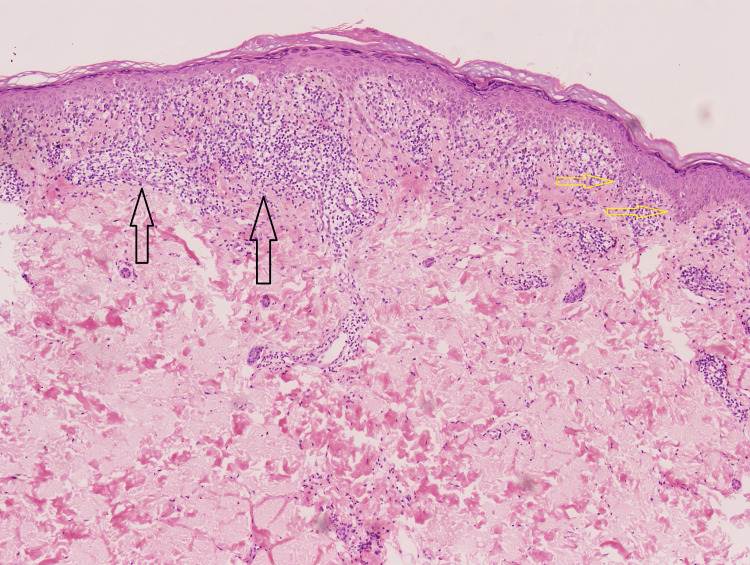
Microscopic appearance of the skin biopsy revealing a band-like lymphocytic infiltrate on the dermal-epidermal junction and saw-tooth rete ridges (H&E, 10x). H&E: hematoxylin and eosin Black arrows: band like lymphocytic infiltrate Yellow arrows: saw-tooth rete ridges

**Figure 8:**
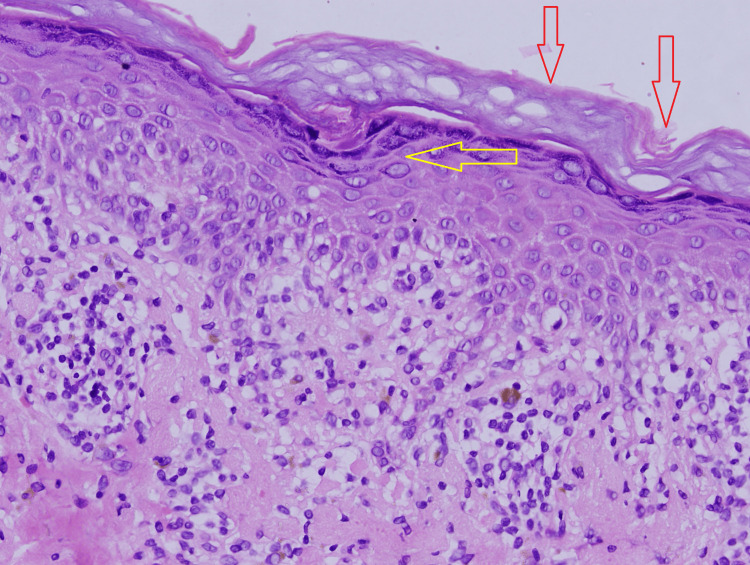
Microscopic appearance of the skin biopsy showing focal vacuolar changes along with hyperkeratosis and hypergranulosis (H&E, 40x). H&E: hematoxylin and eosin Red arrows: hyperkeratosis Yellow arrow: hypergranulosis

**Figure 9:**
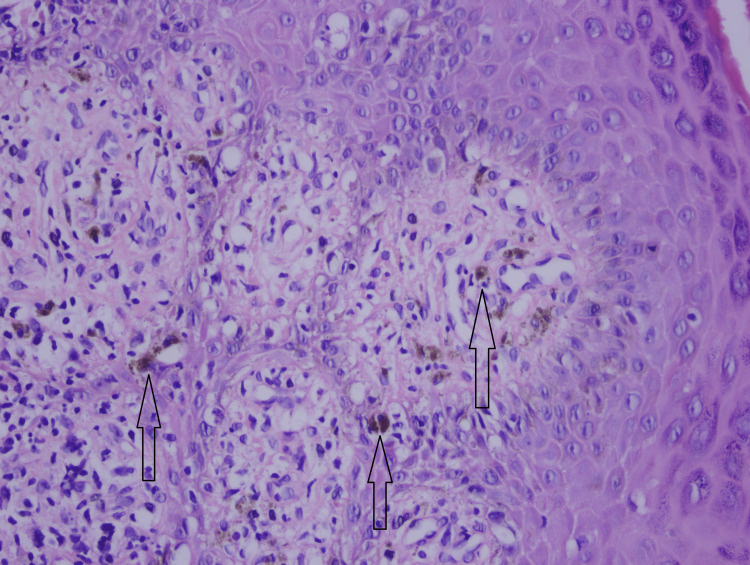
Microscopic appearance of skin biopsy showing scattered pigmentary incontinence with melanophages (H&E, 40x). H&E: hematoxylin and eosin Black arrows: pigmentary incontinence with melanin containing macrophages

Two shave biopsies were taken from two sites to confirm the diagnosis. Light microscopic examination of the skin biopsy from the right forearm revealed hypekeratosis, hypergranulosis, and saw-tooth rete ridges of the surface epithelium. Focal vacuolar changes and band-like lymphocytic inflammatory cell infiltrate were observed at the dermal-epidermal junction (Figures 7, 8). The second biopsy was taken from the axilla, which revealed band-like interface lymphohistiocytic inflammation with scattered pigmentary incontinence with melanin-containing macrophages on the superficial dermis (Figure 9). 

The histopathology findings were correlated with the clinical findings in our patient, and a diagnosis of lichen planus with multiple variants was confirmed.

The patient was started on prednisolone at 30 mg per day for 30 days, followed by a slow taper and the introduction of mycophenolate mofetil (MMF), initially 500 mg twice daily, followed after a few weeks by an increase to 1 gram twice daily, accompanied by topical therapy with clobetasol propionate ointment alternating with tacrolimus 0.1% ointment on a biweekly basis. After a couple of weeks on the treatment plan, the patient began to show significant improvement in all lesions. A complete blood count (CBC), liver function test (LFT), and renal function test (RFT) were all performed routinely throughout the period the patient was receiving mycophenolate mofetil.

## Discussion

The term lichen planus was taken from the Greek word "lichen," which means "tree moss," and the Latin word "planus," which means "flat," thus describing the cutaneous lesions of lichen planus [4]. Lichen planus has been described over the years as a chronic systemic inflammatory condition without a fully understood pathogenesis. But recent studies have shown that lichen planus can be described as a T-cell-mediated autoimmune disease, in which cytotoxic CD8+ T-cells are recruited into the skin and subsequently lead to an interface dermatitis [2,5].

Many subtypes and rare variants of lichen planus exist, which can have significantly different presentations from each other and from the classic form. Furthermore, each of those main subtypes could be further subdivided into smaller categories (e.g., the many forms of oral lichen planus). The major subtypes include annular, bullous, pigmentosus, atrophic, follicular, linear, hypertrophic, actinic, exanthematous, oral, genital, lichen planus/lupus erythematous overlap, pemphigoides type, flexural, psoriasiform, and nail lichen planus [5,6].

Typical or classic cutaneous lichen planus findings Include erythematous to violaceous flat-topped papules and plaques, which tend to be sharply defined and somewhat shiny. Wickham's striae can sometimes be noticed with the naked eye, but are best viewed by dermoscopy [5]. Hypertrophic lichen planus is characterized by the development of hyperkeratotic, thick, pruritic purplish or yellow-greyish papules and plaques, commonly involving the lower extremities, particularly the anterior lower legs [5,6]. Bullous lichen planus forms vesicles and bullae filled with clear or pale-yellow fluid that will develop within the plaque. Typically, blisters do not occur on normal skin, and if present, lichen planus pemphigoides should be suspected. Annular lichen planus usually presents with annular violaceous papules and plaques with a thin elevated border, and a depressed often hyperpigmented center [5]. Annular lichen planus does not cause as much pruritus as other subtypes, and patients are often asymptomatic. It typically involves the axilla, groin, extremities, and male genitalia [7]. Finally, lichen planus pigmentosus is one of the rarer subtypes that is mostly seen in sun-exposed or flexural areas. It presents as hyperpigmented macules, which may or may not have a grayish hue and tend to be distributed in a patchy, follicular, or blaschkoid pattern [7].

On histopathologic examination, classic lichen planus lesions show a linear band-like infiltrate of mostly lymphocytes, accompanied by acanthotic saw-tooth-shaped rete ridges and circumscribed hypergranulosis with an orthokeratotic hyperkeratosis. Apoptotic cells, termed colloid and civatte bodies, are usually the earliest histologic features. Annular lichen planus presents with similar features to classic lichen planus, when the elevated border is biopsied, it will show features of resolved lichen planus, particularly pigment incontinence and epidermal atrophy. The latter feature, if prominent, is often designated "atrophic lichen planus." The hypertrophic variant shows a more pronounced epidermal hyperplasia, often mistaken for a pseudoepitheliomatous hyperplasia or even a cutaneous malignancy, along with similar dermal features to classic lichen planus. Bullous lichen planus, in addition to features of classic lichen planus, shows a subepidermal blister overlying an intense band-like infiltrate of lymphocytes and many apoptotic keratinocytes. Lichen planus pigmentosus usually presents histologically with non-distinctive features, which usually include minimal interface dermatitis and prominent pigment incontinence. These features can be impossible to distinguish from other dermatoses such as erythema dyschromicum perstans or late stages of fixed drug eruption.

In most cases, cutaneous lichen planus resolves spontaneously within one to two years. Therefore, most treatment modalities aim to control pruritus and hasten the resolution of the lesions, although residual hyperpigmentation is quite common. There are numerous treatment options available, including topical, intralesional, and systemic corticosteroids. Other options include griseofulvin, hydroxychloroquine, metronidazole, azathioprine, systemic retinoids, phenytoin, dapsone, cyclophosphamide, interferon alpha-2b, cyclosporine, tetracycline, and phototherapy.

Systemic treatment had to be considered in such a case of severe widespread lichen planus not responding to topical treatment. Systemic steroids will indeed be successful in reducing pain and inflammation, but due to the rebound and relapse risk, long-term corticosteroid use must be avoided. Therefore, mycophenolate mofetil was added to the regimen to help achieve full remission and long-term maintenance since it has a favorable side effect profile and because the authors have had previous successful experiences with using the combination of systemic steroids and mycophenolate mofetil. 

Of note, hypertrophic, ulcerative, and mucosal lesions of lichen planus are considered potential premalignant conditions. Fortunately, the incidence of squamous cell carcinoma in these variants is approximately 1%, yet close follow-up for patients and routine examination of all lesions are strongly recommended to ensure early detection and treatment [3].

## Conclusions

As per our knowledge, this is only the second case report to describe a patient with five forms of lichen planus, and the first to describe one where all five subtypes are cutaneous and non-mucosal. Lichen planus is a versatile skin condition with many different forms, and recognition of all these forms, along with their histologic features, treatment options, and potential complications, is of utmost importance.
